# The role of financial inclusion and technological innovation in stimulating environmental sustainability in the European countries: A new perspective based on load capacity factor

**DOI:** 10.1016/j.heliyon.2024.e39970

**Published:** 2024-11-07

**Authors:** Ahmed Samour, Riza Radmehr, Ernest Baba Ali, Samira Shayanmehr, Elvis Kwame Ofori, Jana Ivanič Porhajašová, Mária Babošová, Miroslava Kačániová, Stephen Kelechi Dimnwobi

**Affiliations:** a Accounting Department, Dhofar University, Salalah, Sultanate of Oman; b Department of Agricultural Economics, Oklahoma State University, USA; c Department of Agricultural Economics, Ferdowsi University of Mashhad, Iran; d Department of Management Science and Engineering, School of Management Engineering, Zhengzhou University, China; e Slovak University of Agriculture in Nitra, Faculty of Agrobiology and Food Resources, Institute of Plant and Environmental Sciences, Slovakia; f Slovak University of Agriculture in Nitra, Faculty of Horticulture and Landscape Engineering, Institute of Horticulture, Slovakia; g University of Economics and Human Sciences in Warsaw, School of Medical and Health Sciences, Okopowa 59, Warszawa, 01 043, Poland; h Department of Economics, Nnamdi Azikiwe University, Awka, Nigeria and Strategy, Execution and Evaluation (SEE) Office, Awka, Nigeria; i Department of Agricultural Economics, University for Development Studies, Tamale P.O. Box TL1350, Ghana

**Keywords:** Environmental sustainability, LCF, Financial inclusion, MMQR, European countries

## Abstract

Given the alarming level of climate change, policymakers across the globe are seeking strategies to mitigate environmental pollution to achieve sustainable development. In this context, renewable energy and technological advancements have emerged as an effective way to lower pollution and attain sustainable development. This study evaluates the effect of financial inclusion, technological innovation, and renewable energy on the load capacity factor (LCF) in European countries from 2004 to 2018. LCF is considered the most comprehensive indicator of ecological sustainability, combining both the biocapacity factor and ecological footprint. Hence, the present work fills the literature gap by exploring, for the first time, the effect of financial inclusion on the LCF. Applying the advanced Method of Moment Quantile Regression (MMQR), the study demonstrates that technological innovation and economic growth have adverse effects on LCF while renewable energy and financial inclusion promote LCF. The study indicates that technological innovation and economic growth undermine ecological excellence in European nations while green energy and financial inclusion enhance it. Moreover, the findings of the causality analysis reveal a causal association between financial inclusion, renewable energy, and LCF. Our study recommends prioritizing financial inclusion alongside investments in renewable energy to enhance ecological sustainability.

## 1 Introduction

Globally, sustainable development is a strategic goal that both advanced and developing nations are striving to achieve, but reports indicate that most economies struggle to synergize environmental preservation and economic growth [[1], [2], [3], [4]]. Environmental degradation is the main barrier to sustainable growth and as such clean energy is now getting increased attention as the world begins to suffer from the consequences of global warming, such as rising temperatures and sea levels, excessive droughts, melting glaciers, and altered rainfall patterns [[5], [6], [7]]. The urgency of addressing this climate crisis is underlined by several international commitments like the Paris Agreement, Sustainable Development Goals (SDGs), and the UN Climate Change Conference [[3], [4], [5],^8^]. The utilization of modern technology might hasten the shift to cleaner energy sources, thereby making the battle against climate change easier [9]. Energy security and clean energy technology are crucial for moving towards a world economy that is sustainable [10]. The move from nonrenewable (fossil fuel) energy to renewable energy generation has benefited greatly from clean energy technology [11,12]. These innovations have boosted energy output from renewable energy sources, including solar, wind, and nuclear power plants [13]. Although clean energy technologies have several advantages, the present rate of adoption may not be sufficient to ameliorate the consequences of climate change and reach net-zero target [4,5,14]. Climate change and a slow economic progress plagued by extreme wealth disparity are the two biggest problems the post-COVID world must deal with [15]. Therefore, it is necessary to look for answers that are not only practical but also all-encompassing [2,16].

The poorest people on the planet are anticipated to be the main victims of climate change, even if its origins may be traced to the lifestyles of the rich (advanced economies) [17]. Such a description best captures the European countries, which represent one of the highest contributors to the ecological disruption associated with economic growth [E.U. 18, 19]. As such, there is a need to interrogate further pathways which can be used to curb such growth patterns. The transition to a low-carbon economy necessitates substantial capital allocation towards renewable energy projects and cutting-edge carbon mitigation technologies [[18], [19], [20]]. Integrated Assessment Models (IAMs) consistently emphasize the critical role of negative emission technologies, particularly Bioenergy with Carbon Capture and Storage (BECCS), in achieving net-zero emissions scenarios. However, the implementation of BECCS and similar technologies requires significant financial resources, estimated at $138–229 billion annually by 2050 [21].

The magnitude of BECCS-related expenditures serves as a paradigmatic example of the broader financial challenges inherent in green energy transitions [22]. Initial capital outlays for renewable energy infrastructure - encompassing wind, solar, and hydroelectric power generation - are comparably substantial. These costs are not limited to facility construction and installation but extend to the integration of these energy sources into existing power grids, often necessitating significant upgrades to accommodate the variability and distributed nature of renewable generation [23]. Furthermore, while the operational costs associated with renewable energy systems are generally lower than those of fossil fuel-based alternatives [24], they still represent a significant financial burden. In the specific case of BECCS, operational expenses are further amplified by the requisite continuous biomass supply chain and the energy-intensive nature of carbon capture and storage processes [25].

Hence, financial development emerges as a pivotal factor in the transition to a low-carbon economy, serving as the essential fuel for both climate mitigation and adaptation strategies - building resistance to the damaging effects of climate change. The substantial capital requirements for renewable energy projects and negative emission technologies underscore the critical role of robust and inclusive financial systems. These systems act as conduit for mobilizing the necessary resources, facilitating investments, and distributing risks associated with large-scale environmental initiatives [1]. Despite the recognized importance of financial development in facilitating the transition to a low-carbon economy, several significant research gaps and limitations persist, necessitating further investigation. These include inadequate consideration of heterogeneity in financial systems [26], limited understanding of long-term impacts [27], insufficient exploration of policy interactions [28], and the need for more sophisticated risk assessment models for green technologies [29]. Additionally, there are gaps in our understanding of SME financing in the clean technology sector [30], the role of digital finance in accelerating green technology adoption [31], and strategies for managing transition risks associated with stranded assets [32]. The lack of standardized metrics for quantifying the impact of financial development on climate change mitigation, limited application of behavioural finance principles to green investments, and insufficient research on the resilience of green finance mechanisms during economic downturns further underscore the need for comprehensive studies [33]. This research aims to address some of these gaps by examining the relationship between financial inclusion, technological innovation, and environmental sustainability through the novel application of the load capacity factor.

Linking this to prior literature, econometric evaluations of financial development-environmental footprint nexus reveal a dichotomous pattern, characterized by both eco-efficiency gains and potential rebound effects in resource consumption [29,[34], [35], [36], [37], [38]]. On the one hand, financial inclusion makes it simpler for enterprises and people to obtain beneficial and cheap financial plans, increasing the viability of green technology investments [39]. According to Ref. [40]) and [1], the provision of inexpensive financial services and commodities may greatly encourage the usage of clean and renewable technologies. The financial system may promote the uptake of these technologies by reducing the cost of renewable energy investment for individuals, businesses, and governments. The financial system is crucial to the growth and advancement of the renewable energy industry since it makes acquiring finance and project financing easier [39,41]. This might include investing in new technology, building infrastructure, and developing new energy markets. By reducing capital costs, the financial system can increase the competitiveness of renewable energy, making it more accessible and less expensive for a larger variety of users. The use of financial instruments like carbon credits and green bonds may also aid the transition to a low-carbon economy [42,43]. As a means of boosting accessibility and adoption of improved environmental practices that lessen climate crisis, inclusive financial systems positively affect the environment [36]. In poor communities, farmers might not have access to capital or credit to invest in green energy technologies, such as solar energy microgrids, which are economical and mitigate a lot less CO_2_ than burning coal; promoting financial inclusion is very critical [44].

On the other hand, increased financial services accessibility promotes and supports manufacturing and industrial activity, which may result in greater CO_2_ emissions, contributing to global warming [45]. Furthermore, increasing financial inclusion enables individuals to purchase energy-intensive consumer goods like vehicles, refrigerators, and air conditioners, the usage of which presents a serious environmental risk due to higher greenhouse gas (GHG) emissions. In addition to fostering economic activity, inclusive financial systems also boost demand for dirty energy sources, which raises GHG emissions [46,47]. These contradictions make policy implementation difficult, hence necessitating further work to help settle the debate on financial inclusion and ecological sustainability.

Another major gap in the literature is a better assessment of the variables driving ecological sustainability. Most research has concentrated on total energy consumption and economic expansion. However, using either one or the other may not adequately convey ecological imbalance. The emphasis of economies has shifted to clean energy and environmentally friendly technology to achieve sustainable economic development [48]. Ecological footprints (EF), which show a sustainable ecosystem, are also often employed to assess environmental pollution. EF, on the other hand, portray anthropogenic strain on the ecosystem and connects human consumption with the biosphere's ability to regenerate. As a result, we must concentrate on examining how the use of renewable energy, innovation, and financial inclusion relate to environmental risks. In improving the proxies used to study ecological sustainability, this study employs the load capacity factor. The load capacity factor (LCF) is a key indicator for assessing the overall state of environmental sustainability. It is a crucial metric in environmental sustainability, quantifying resource utilization relative to an ecosystem's maximum sustainable threshold. This concept provides a tangible measure of human impact on natural systems, resonating with themes in sustainability literature, including Donella Meadows, Randers [49] ' “The Limits to Growth" and the Brundtland Commission's “Our Common Future" (1987) [50]. Additionally, the LCF offers a quantifiable and comparable metric across sectors, enables the setting of clear targets and limits, and helps identify overexploitation before irreversible damage occurs. By simplifying complex ecological concepts, the LCF aids policymakers and businesses to make informed resource management decisions, aligning with [[51], [52], [53], [54]] reports and recommendations.

Specifically, the LCF acts as a general indicator of ecological sustainability: (i) It is specifically created to show how consumption, supply, and waste interactions affect environmental quality [55,56], (ii) It might be used as a tool to assess various horizons and the level of human stress on the planet [57]. In addition, it maximizes the return on investment for project activities in society and improves citizen welfare [58]. It also aids individuals, social leaders, and nations in defining how their behaviour affects the environment on a global scale. The LCF, which addresses a variety of natural resource demands and supplies, has the potential to expand the conversation about a sustainable environment beyond concerns about global warming and climate change [58]. It offers a framework for prioritizing tasks, identifying various strategic action plans, and evaluating performance in relation to stated objectives [59]. Biocapacity and ecological footprint are used to calculate LCF. When LCF is greater than 1, the environment is sustainable; when it is less than 1, it is not. As such the LCF has lately caught the curiosity of academics due to its obvious relevance and application of an ecological viewpoint to sustainability concerns. The following discoveries to the ever-expanding body of knowledge are what we want to make.

Despite the potential benefits, a significant portion of the population remains excluded from financial inclusion and technology, highlighting a disparity between the availability of finance and its impact on the environment and the pursuit of Sustainable Development Goal 13. Again, the literature lacks a sufficient discussion on the interconnection between these areas and the challenges they pose to achieving SDG 13 and improving the ecological footprint. This study adds some original insights to the field.

• This study contributes to the ongoing discourse led by the World Bank, emphasizing the importance of financial inclusion as a catalyst for achieving Sustainable Development Goals (SDGs) in developing and impoverished nations [60], with a particular focus on improving the environment and aligning with SDG 13. The insights presented herein offer valuable information for national and global policymakers, enabling them to grasp the complexities associated with the rapid expansion of financial services, its accessibility to marginalized populations, and the inherent risks involved in digital inclusion through technology.
• Moreover, for scholars and researchers, this study expands upon the growing body of literature on financial inclusion, aiming to propose effective solutions for fostering sustainable development across the globe. The ideas put forth in this article call for further collaborative research, seeking a deeper comprehension of the interplay between financial inclusion, environmental preservation, and the associated risks and alternative models within this domain.
• Additionally, this paper contributes to the limited existing studies that explore the role of financial innovation in promoting ecological stability. By drawing insights from this article, our understanding of the functions performed by Fintech companies can be enhanced, while regulators can gain valuable insights into the intricate connection between Fintech, financial inclusion, and the stability of financial systems.

Following the introduction, section 2 shows an overview of the related empirical literature. Section 3 presents the data and the methodology, and Sections 4, 5 show the empirical outcome and conclude the study, respectively.

## 2 Literature insights

Numerous theories can be utilized in explaining how technological innovation (TI) and financial inclusion (FI) influence ecological safety. Innovation Diffusion Theory (IDT) propounded by Everett Rogers is one such theory. IDT emphasizes that innovative concepts could be endorsed across different population segments of a country, and this endorsement could influence the way the society operates. 10.13039/100017146FI is critical in supporting the utilization of green devices because it provides the needed financial backing and reduces the economic risks that are usually associated with such undertakings [61]. Increased access to credit will propel firms and individuals to obtain and utilize this low-carbon device, thereby overcoming the usual challenges that early adopters face and enhancing a wider uptake of sustainable devices and practices [62]. TI, on the other hand, can offer unique strategies and concepts that are pro-environment, which may include green appliances or tools and contemporary waste-management techniques [63]. When these technologies made possible by FI expands, their utilization is broadly accepted, thus ensuring that sustainability drive is very feasible for the wider population [64]. The spread of these sustainable practices and technologies could spark a snowball effect that encourages a broader acceptance of green standards [62]. It is also possible that the early adopters of this invention and sustainable practices could encourage others to tap into these measures, which will eventually lead to a paradigm switch towards eco-conscious behaviours [61].

Relatedly, the **Ecological Modernization Theory** (EMT) provides the hypothesis for explicating the nexus between FI, TI and ecological performance. The model avers that technological advancements, specifically eco-friendly technologies could aid industries to minimize the pollution levels emanating from their operations [65]. The theory stresses that the integration of innovations into firm's production techniques boosts ecologically friendly endeavours and reduces environmental decay [66]. 10.13039/100017146FI is essential in driving sustainable endeavours as it expands accessibility to credit, thereby enhancing enterprises' and individuals' capacity to fund low-carbon projects [63,67]. By energizing the broad uptake of these low-carbon devices, FI stimulates ecological balance [65]. In conclusion, EMT highlights how technology and finance when aligned with sustainability objectives, simultaneously spur economic and ecological quality.

The review of prior works is segmented into two parts. The first strand is on the nexus between FI and the environment while the other stream is on TI and ecological preservation. Using data from G7 economies, Xin and Xie [68] explore the nexus between FI and the ecological quality between 2000 and 2020 and reveal that emissions in these economies are accelerated by FI. In the E7 bloc, Qin et al. [69] find that FI suppresses ecological quality, particularly at the lower quantile grid while it has no significant correlation at the upper quantile grid. Ahmad and Satrovic [10] confirm in 7 OECD economies that FI is not beneficial to ecological quality which also aligns with another study in 23 OECD economies authored by Zaidi et al. [70]. This is also related to the outcome obtained in Eurozone between 1995 and 2018 by Fareed et al. [71] and in China by Dong et al. [72] from 2004 to 2018 revealing that FI retards ecological stability. Chaudhry et al. [73] also document that FI decelerates ecosystem sustainability in selected Islamic nations. Le et al. [37] make a similar conclusion for 31 Asian economies from 2004 to 2014. In contrast, some studies find that the environment responds favourably to the increase in FI. For instance, Yuan and Alharthi [74] appraise the nexus between FI and carbon emissions in selected 20 Asian nations from 1990 to 2020 and document that FI is pro-environment. Focusing on one of the most polluted economies of the world (China), Shahbaz et al. [75] demonstrate that FI boosts ecological sustainabilty by reducing pollution levels. This is also supported by a study of 15 top carbon emitters by Usman et al. [76] underlining the criticality of 10.13039/100017146FI in promoting ecological balance. Hodžić et al. [77] report using data from 2004 to 2019 in European economies that FI lessens carbon emissions, especially in the upper quantiles.

Away from studies on FI and the environment, research attention has been directed at the effect of TI and the environment, albeit with mixed outcomes. Chen et al. [78] disclose using Bangladesh's data to show that TI benefits the ecosystem by reducing carbon emissions. This is also related to the outcome established by Ahmad et al. (2023) [79] in China using carbon emission as a surrogate for ecological quality. Feng et al. [80] establish that TI is favourable to China's environment using EF to proxy ecological balance. In a broader sample (BRICS), Adebayo et al. [81] report that ecological degradation is mitigated with TI showing the enhancing influence of TI on ecosystem protection. In the context of G-10 economies, Guan et al. [82] find that ecological strain is ameliorated with TI, indicating that TI is vital in preserving the environment. In 22 emerging nations, Ahmad et al. [83] validate the importance of TI in ecological preservations. The study of Destek and Manga [84] in emerging economies is a bit different as the environmental effect of TI is contingent on the ecological indicator deployed. When carbon emission is used, TI lowers the emission level but the effect of TI on alternate ecological measure (EF) is negative but not significant. Zhao et al. [85] made a similar observation in 5 emerging economies using only LCF as the ecological indicator. They find that TI has a negative though insignificant effect on LCF. Ofori et al. [39] also support this outcome for Eurozone using carbon emission to proxy ecological quality. This suggests that the influence of TI on the environment depends on the environmental measures used. On the flip side, Ali et al. [86] disclose that the European Union's environmental quality is degraded by TI. Kartal and Pata [87] apply data from China between 1990 and 2020 to discover that TI is unfavourable to the ecosystem. This suggests that the Chinese environment is damaged with increased advancements in technology. Table 1 provides an overview of these studies.

**Table 1:** Synopsis of prior studies reviewed.

Authors	Country(s)	Period	Method	Finding
**(a) Financial inclusion (FI) - environmental quality**				
Xin and Xie [68]	G7 countries	2000–2020	non-parametric method	FI→ CO_2_ (−)
Shahbaz, Li [75]	30 Chinese provinces	2011–2017	benchmark regression	FI→ CO_2_ (−)
Fareed, Rehman [71]	27 European countries	1995–2018	panel quantile regression	FI→ EF (+)
Zaidi, Hussain [70]	23 OECD countries	2004–2017	CS-ARDL	FI→ CO_2_ (+)
Usman, Makhdum [76]	15 highest emitting countries	1990–2017	AMG	FI→ EF (−)
Le, Le [36]	31 Asian countries	2004–2014	DKSE	FI→ CO_2_ (+)
Qin, Raheem [69]	E7 countries	2004–2016	panel quantile regression	FI→ CO_2_ (+)
Dong, Dou [72]	China	2004–2018	Spatial econometrics model	FI→ CO_2_ (+)
Yuan and Alharthi [74]	20 Asian developing nations	1990–2020	CS-ARDL	FI→ CO_2_ (−)
Chaudhry, Yusop [73]	OIC countries	2004–2018	DCCE	FI→ CO_2_ (+)
**(b) Technology innovation (TI) - environmental quality**				
Chen, Rahaman [78]	Bangladesh	1972–2020	ARDL	TI→ CO_2_ (−)
Adebayo, Ullah [81]	BRICS countries	1990–2019	CS-ARDL	TI→ CO_2_ (−)
Ahmad, Youjin [79]	China	1982–2018	ARDL	TI→ CO_2_ (−)
Ofori, Li [2]	European countries	2002–2017	Panel spatial model	TI→ CO_2_ (≠)
Baba Ali, Radmehr [86]	European countries	1996–2018	quantile regression	TI→ CO_2_ (+)
Zhao, Samour [85]	BRICS-T nations	1990–2018	CS-ARDL	TI≠ LCF
Feng, Chong [80]	China	2011–2019	OLS, 2SLS	TI→ EF (−)
Guan, Rani [82]	G-10 countries	1995–2019	CS-ARDL	TI→ EF (−)
Destek and Manga [84]	BEM economies	1995–2016	CUP-FM, CUP-BC	TI→ CO_2_ (−) TI≠ EF
Ahmad, Jiang [83]	emerging economies	1984–2016	CS-ARDL	TI→ EF (−)

DKSE: Driscoll-Kraay standard errors; DCCE: Dynamic Common Correlated Effects; BEM: Big emerging markets; CS-ARDL: Cross-sectional autoregressive distributed lag; AMG: Augmented mean group; ARDL: Autoregressive distributed lag; OLS: Ordinary least square, 2SLS: Two-stage least squares; CUP-FM: Continuously-updated and fully-modified; CUP-BC: Continuously-updated and bias-corrected; EF: ecological footprint; ≠ insignificant effect; (+): positive and significant effect; (−): negative and significant effect.

The survey of the literature has shown that most of the related studies have either utilized carbon emissions or ecological footprint as ecological quality measures. These ecological indicators only evaluate the demand dimension of the environment thereby ignoring the supply side. To rectify this predominant issue in the literature, this study utilizes LCF which provides more holistic measures of the environment by merging both the supply and demand components of the ecosystem. Another notable observation from the literature is that most studies have primarily utilized the mean-based technique assuming that relationships between variables are linear but this is not always the case in the real world. To resolve this constraint, we apply the MMQR which supports the assessment of the heterogeneous influence of regressors across several distributions of the data. Lastly, research attention on the influence on either TI and environment or FI and environment has been limited in the panel of European nations. This is surprising given that these economies are leading innovative economies of the world as well as possessing substantial environmental and economic influence. Our study bridges this lacuna by pioneering the assessment of FI and TI on LCF in European nations in a single framework. This study will pinpoint the variables that either enhance or undermine ecological sustainability in these economies.

## 3 Data and methodology

### 3.1 Data

We scrutinize the impact of technology innovation, financial inclusion, and economic growth on the load capacity factor in European countries. To achieve our research objectives, we used annual data spanning from 2004 to 2018. The time frame of this study is selected based on the data availability of the explored variables. The tested countries (Austria, Belgium, Czech Republic, Croatia, Republic of Cyprus, Denmark, Estonia, Finland, France, Greece, Germany, Hungary, Italy, Latvia, Luxembourg, Lithuania, Norway, Netherlands, Portugal, Poland, Romania, Spain, Slovakia, Switzerland and Sweden). We dropped some countries due to data unavailability.

In the examined model, we proxy ecological quality by load capacity factor [88]. We proxy renewable energy using the share of this consumption to the total energy use [85]. We used GDP/per capita (Constant 2015 US dollars) as a proxy of economic growth [89]. Likewise, we used the number of ATMs per “100,000″ adults as an indicator of financial inclusion [90]. We proxy technology innovation by patents (Residents, nonresidents) [91]. The description of the mentioned variables and sources of data is presented in Table 2.

**Table 2:** Variables description and sources of applied data.

Variable	Variable Description
LCF	Load capacity factor as a proxy to evaluate the ecological sustainability by dividing biocapacity (per capita) on the EF (Global hectares per person)
REC	renewable energy share to the total utilization of energy
GDP	Per capita (Constant-2015 US dollars)
lnFI	Number of ATMs per 100,000 adults
lnTI	Patents (Residents, nonresidents)

### 3.2 Model specification

The current investigation examines the impact of technology innovation, financial inclusion, economic growth, and renewable energy consumption on LCF in European countries. Following Pata and Samour [88], we have formulated the empirical models as follows:

(1) $$LCF_{it}=f\;\left(GDP_{it},REC_{it},FI_{it},TI_{it}\right)$$

In equation (1), $LC_{\text{it}}^{F}$ depicts load capacity factor. ${\text{GD}}_{\text{it}}^{P}$, ${\text{RE}}_{\text{it}}^{C}$, $F_{\text{it}}^{I}$, $T_{\text{it}}^{I}$ present economic growth, renewable energy consumption, financial inclusion and technology innovation, respectively. To avert heteroscedasticity, the selected variables are transformed into natural logarithmic forms. The econometric model is shown as follows in equation (2):

(2) $$\ln\;LCF_{it}=\delta_{0}+\ln\;\delta_{1}GDP_{it}+\ln\;\delta_{2}REC_{it}+\ln\;\delta_{3}FI_{it}+\ln\;\delta_{4}TI_{it}+e_{t}$$

In the above equation, ln depicts the logarithm, $\delta_{1}$ …. $\delta_{4}$ are the coefficients of the selected variables, t means the time, i stands the country, and $e_{t}$ denotes error term [68].

### 3.3 Econometric strategies

#### 3.3.1 Cross-section dependency (CD) and. Unit root tests

We employ some preliminary techniques to confirm that the tested empirical model is reliable. First, we use [13]) cross-sectional dependence (CSD) assessment to check the cross-sectional issue, which is structured in the following equation:

(3) $$\text{CD}=\sqrt[{.}]{\frac{2T}{N\left(N-1\right)}}\left(\sum_{i=1}^{n-1}.\sum_{K=i+1}^{N}p_{IK}\right)\;N\left(0,1\right)I,K=1,2,3,\ldots \ldots \ldots N$$

In equation (3), N depicts the cross-sections; T indicates time.

Next, we use first and second-generation tests to check the stationary issue. In this context, the study uses the cross-sectionally augmented Im, Pesaran, & Shin (CIPS) suggested by Ref. [92] to evaluate the stationary of the focused variables. Next, we use slope heterogeneity assessment to assess the heterogeneity issue in our empirical model. In null hypothesis assumes the absence of homogeneity, while the alternative hypothesis suggests the existence of homogeneity. The slope heterogeneity assessments are structured in equations (4), (5):

(4) $${\tilde{\Delta }}_{SH}={\left(N\right)}^{\frac{1}{2}}{\left(2k\right)}^{-\frac{1}{2}}\left(\frac{1}{N}\tilde{S}-k\right)$$

(5) $${\tilde{\Delta }}_{ASH}={\left(N\right)}^{\frac{1}{2}}({\frac{2k(T-k-1}{T+1})}^{-\frac{1}{2}}\left(\frac{1}{N}\tilde{S}-2k\right)$$

#### 3.3.2 Panel co-integration test

Before estimating the long-run interconnections3, the co-integration link amid the variables should be validated. We use the Kao test, Pedroni test, and Westerlund tests of co-integration to evaluate co-integration among technology innovation, financial inclusion, economic growth and load capacity factor in European countries. The null hypothesis (H0) implies the absence of co-integration amid the focused variables. While the alternative hypothesis (H0) suggests the existence of co-integration among the selected variables.

#### 3.3.3 MMQR test

We employed the novel approach of MMQR as introduced by Machado and Santos Silva [93] to detect the causes of ecological quality in European countries. This method is a superior technique since it can display the impacts of all the focused variables on load capacity factor at lower, median, and higher load capacity factor levels. Unlike the traditional quantile techniques, MMQR provides consistent and efficient outcomes if there are multicollinearity and endogeneity issues in the tested models. The random variable conditional quintile of Qy(τ|X) is formulated in equation (6):

(6) $$Y_{it}=\alpha_{it}+X_{it}^{΄}\;\beta +\left(\delta_{i}+Z_{it}\;\gamma^{,}\right)\mu_{it}$$

The probability of $\delta_{i}+Z_{it}\;\gamma^{,})$ >0 and equal to 1 while Y, β, andδ are the estimated parameters. The fixed effects of the employed model are evaluated by and δ. Z represents the differentiable transformation of J vector of X and is structured in the following equation:

(7) $$Z_{it}=Z_{it}\left(X\right),I=1,2\ldots .,J$$

In equation (7), $X_{it}^{΄}$ is identically and separately disposed of across fixed (i) and time (t). $\mu_{it}$ is standardized to realize the conditions moment. This can be structured as in Eq. (5):

(8) $$Q_{y}\left(\tau X_{\text{it}}\right)=\left(\alpha_{\text{it}}+\delta_{i}q\left(\tau \right)\right)+X_{\text{it}}^{΄}\;\beta +Z_{\text{it}}\;\gamma ΄q\left(\tau \right)$$

In equation (8), $X_{it}^{΄}$ depicts vectors of the independent variables' vectors (GDP, REC, FI and TI) in the natural logarithms $(Q_{y}\left(\tau X_{\text{it}}\right)$ depicts the quintile distribution of the LCF conditional on the location of focused explanatory variables. (i) and time (t) fixed effects presented by the scalar coefficient formulated as. $\prime \prime X_{it}^{΄}$ $\alpha_{it}\left(\tau \right)=$ $\alpha_{it}+\delta_{i}q\;\left(\tau \right)\text{.}$ Unlike the traditional statistical fixed least-squares outcomes, the individual impact [10] implies no intercept shift. The τ-the sample quantile is further generated after retrieving the solution by minimizing equation (9):

(9) $$\min_{q}\sum_{\;i\;}\sum_{t}\rho_{\tau }\;\left(R_{it-}\;\left(\delta_{i\;}+Z_{it}^{\prime }\;\gamma \right)\;q\right)$$

#### 3.3.4 Robust estimators

We use the Fixed Effects OLS (FE-OLS) method test with [94] standard errors to offer a baseline empirical investigation for the model specifications. In addition, we use Driscol-Karaay Regression, which has the advantage of taking into consideration the cross-sectional dependency and heteroscedasticity issues. Besides, we use the Dynamic-Ordinary Least Squares(-D-OLS) estimating test proposed by Ref. [95]. The test is protracted through Monte Carlo simulation. Likewise, we use the Fully Modified-OLS (FMOLS) presented by Ref. [96] where the intercepts are unique for each employed series studied. However, FMOLS is considered a reliable estimate when the sample size is small and eliminates the endogeneity problem and serial-correlation between the focused variables. Besides, the DOLS test also solves the endogeneity problem by taking into account the first differences of the leads and lags of the regressors (Yu et al., 2024). This method solves the issue of endogeneity across cross-sectional units. Hence, these methods provide more efficient coefficient estimates [97].

**Fig. 1:**
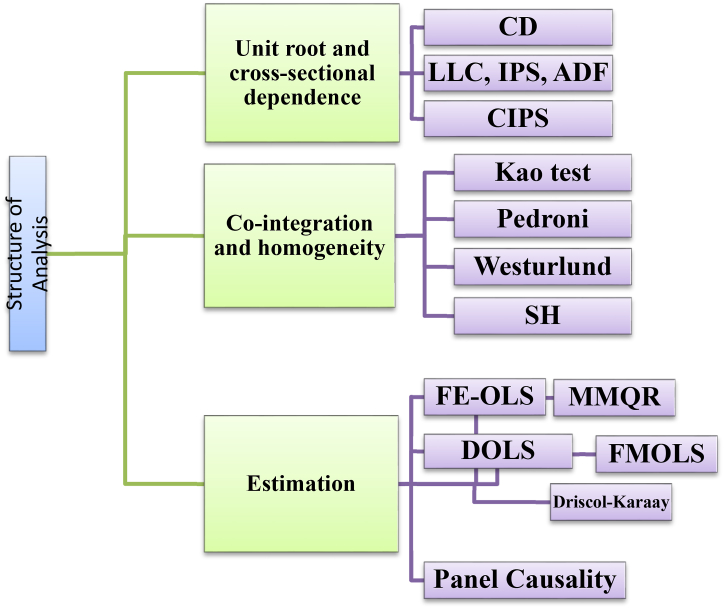
Structure of analysis.

Finally, we use the Dumitrescu and Hurlin causality analysis, as proposed by Ref. [98] to evaluate the causal connection between technology innovation, financial inclusion, economic growth, and load capacity factor in European countries. Fig. 1 shows the structure of the employed analysis.

## 4 Results and discussion

**Fig. 2:**
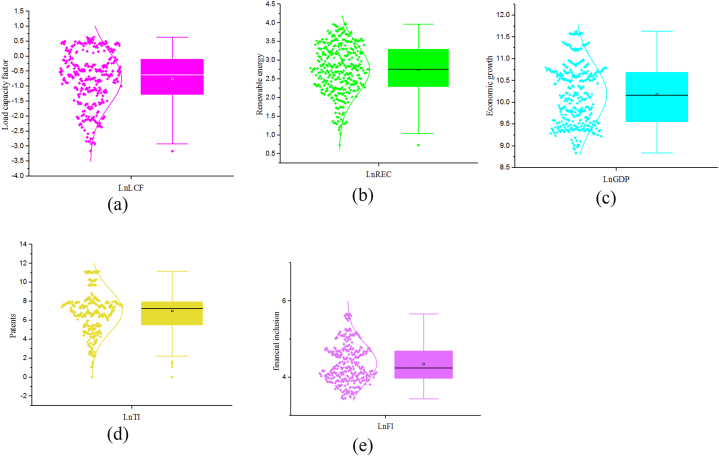
Graphical representation of data. Panels (a), (b), (c), (d), and (e) show the distribution of lnCF, lnREC, lnGDP, lnTI, and lnFI, respectively.

**Fig. 3:**
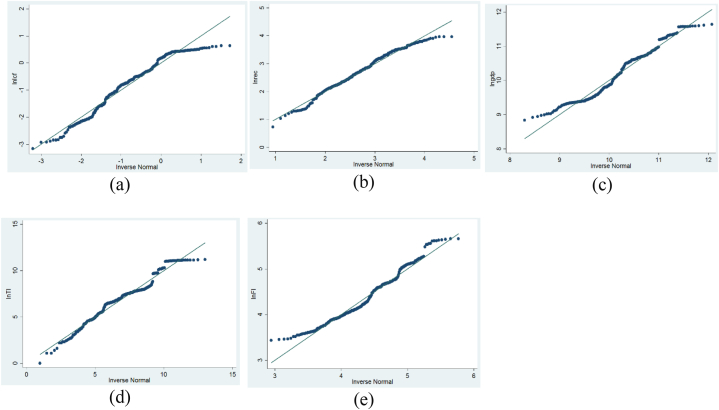
Normality graph of variables.

In the events leading to the statistical estimation of the study results, a battery of preliminary tests was performed to determine the appropriateness of our study variables for the model we intend to employ. First, the distribution of the data was examined using the scatter plot (Fig. 2). The scatter plot only provides a visual inspection of the data distribution without providing detailed information. Thus, from Fig. 2, we can conclude that the data has an almost normal distribution. Relying on this information for our empirical analysis may result in the estimation of biased outcomes. As a result, we continue to test the distribution of the data by applying a series of different normality tests. In assessing the normal distribution using the skewness, the underlying assumption is that the value of the variable must be zero. Results from the skewness show an abnormally distributed dataset (i.e., LCF, REC, and TI have negative deviation, while GDP and FI have positive distribution). Furthermore, the normal distribution using the Kurtosis is investigated. The Kurtosis is used to examine the steepness of the dataset. The underlying assumption is that a value of zero signifies an even steepness of the dataset. The result shows values greater than zero for all the study variables, suggesting that the study's dataset violates the normal distribution assumption. Additionally, the authors performed the Shapiro-Wilk test and Kolmogorov-Smirnov tests to further ascertain the normality. Whereas the former examines the normality vis-sa-vis the significant differences in the data, the Kolmogorov-Smirnov test postulates that the normality is validated with a test value closer to 1. The result for the Shapiro–Wilk shows that all the variables are significant at 1 %, demonstrating an acceptance of the alternate hypothesis. This implies that the study variables have abnormal distribution. Finally, given the test statistic of all the variables under the Kolmogorov-Smirnov test, we conclude that the data have abnormal distribution. To check for the robustness of the normality test results, we used the quantile-quantile normality Normality test (Table 3) to supplement the examination of the data (Fig. 2, Fig. 3). The result failed to confirm the normality of the data as a deviation from the normal distribution line was observed.

**Table 3:** Normality test results.

Variables	Skewness	Kurtosis	Shapiro-Wilk test		Kolmogorov-Smirnov (k) test	
			Statistics	P-value	Statistics	P-value
LnLCF	−0.473	2.472	11.446	0.000	0.813	0.000
LnREC	−0.479	2.951	5.943	0.000	0.094	0.002
LnGDP	0.158	2.063	9.363	0.000	0.084	0.008
LnTI	−0.220	3.177	7.751	0.000	0.093	0.002
LnFI	0.494	2.613	5.915	0.000	0.084	0.009

### 4.1 Slope homogeneity (SH)) and CD results. Panels (a), (b), (c), (d), and (e) show the normality graph of lnCF, lnREC, lnGDP, lnTI, and lnFI, respectively

After performing the normality test, the study proceeded to inspect CD among the study variables (Table 4). The essence of this test is to determine whether or not the CD effect exist among the study variables. In order words, the CD test is used to ascertain the possibility of a variation of any of the covariates in one county due to a disturbance of a similar covariate in another nation. From the test result, it is observed that the variables are statistically significant, indicating that the alternate hypothesis is accepted and that the cross-sectional dependence among the countries of the study is valid.

**Table 4:** Results of CD test.

Variables	CD test	P-value	Cprr	Abs (corr)
LnLCF	23.77	0.000	0.351	0.409
LnREC	60.32	0.000	0.882	0.882
LnGDP	32.27	0.000	0.467	0.655
LnTI	1.86	0.062	0.026	0.440
LnFI	14.87	0.000	0.216	0.562

The test of slope homogeneity is presented in Table 5. The essence of the SH test is to interrogate whether the slopes of two or more regression lines are significantly different from each other. The test is used in statistical analysis to assess the validity of the assumption that the slopes of the regression lines under different conditions are equal. The result implies that there is evidence to support the alternate hypothesis of equal slopes and conclude that the slopes of the regression lines under different conditions are significantly different. In other words, it indicates that the differences in the observed values of the dependent variable between the groups or conditions cannot be explained by chance and that there is a real difference in the connection between the covariates and the main variable for the different groups or conditions.

**Table 5:** Results of the slope homogeneity.

Test	Value	P-value
$\tilde{\Delta }$	5.081	0.000
${\tilde{\Delta }}_{adjusted}$	6.942	0.000

### 4.2 Unit roots results

The test of unit roots is an important pre-estimation procedure, used to determine whether the study covariates are integrated of order I(0), I(1), or both. Conversely, the unit roots test determines whether the statistical properties of the panel data are stationary or vary over time. If the panel data is found to be non-stationary, it may influence the estimation of spurious outcomes. From the results (Table 6), it is observed that stationarity runs through the data, albeit at level and first difference.

**Table 6:** Unit root tests results.

Variables	1st generation test						2nd generation test	
	LLC		IPS		ADF-Fisher		CIPS (zt-bar)	
	Level	Δ	Level	Δ	Level	Δ	Level	Δ
LnLCF	−3.679∗∗∗	–	−2.868∗∗∗	–	95.652∗∗∗	–	−2.776∗∗∗	–
LnREC	−7.599∗∗∗	–	−0.352	−7.544∗∗∗	77.268∗∗	–	−2.145∗	–
LnGDP	−3.225∗∗∗	–	2.966	−5.126∗∗∗	27.572	131.601∗∗∗	−1.614	−2.833∗∗∗
LnTI	−2.364∗∗∗	–	−0.620	−8.153∗∗∗	118.878∗∗∗	–	−1.927	−3.411∗∗∗
LnFI	−0.189	--4.067∗∗∗	1.271	−3.476∗∗∗	163.908∗∗∗	–	−1.573	−2.253∗∗

Note: Δ: First difference. ∗, ∗∗, and ∗∗∗ stants for p-value <10 %, 5 %, and 1 %, respectively.

### 4.3 Co-integration test results

In panel data analysis, the co-integration test is important for determining the existence of long-run connections. We, therefore, employ the panel co-integration test as shown in Table 7. The result shows evidence of co-integration among REC, GDP, TI, FI and LCF variables. This infers the likelihood of long-term nexus among the tested variables.

**Table 7:** Results of the panel co-integration test.

Tests	Statistic	P-value
***Kao test***		
Modified DF t	−2.918	0.001
DF t	−4.045	0.000
Augmented DF t	−1.776	0.037
Unadjusted modified DF t	−6.833	0.000
Unadjusted DF t	−5.760	0.000
***Pedroni test***		
Modified PP t	3.616	0.000
PP t	−11.134	0.000
Augmented DF t	−11.113	0.000
***Westurlund test***		
Variance ratio	−2.510	0.006

Dickey-Fuller: DF; Phillips-Perron: PP.

### 4.4 Long-run estimators results

The current paper deployed a sequence of empirical estimators (FMOLS, DOLS, FE-OLS, Driscol-Karaay Regression) to explore the link between technology innovation, financial inclusion and load capacity factor. The findings of FMOLS, DOLS, FE-OLS and Driscol-Karaay Regression tested are presented in Table 8, Table 9. The estimated outcome from FMOLS, DOLS, and FE-OLS models shows some interesting findings. First, the study reveals a high level of significance among the covariates, that is, whereas about 75 % of the study outcomes are significant at 1 % across the three estimators and the robustness analysis, the remaining 25 % have a 5 % level of significance, indicating a strong reliability of the study results.

**Table 8:** FMOLS, DOLS, and FE-OLS results (LCF dependent variable).

Variables	FMOLS		DOLS		FE-OLS	
	Coeff.	t-stats	Coeff.	t-stats	Coeff.	t-stats
LnREC	0.097∗∗∗	3.971	0.101∗∗∗	3.177	0.120∗∗∗∗	6.66
LnGDP	−0.147∗∗	−1.964	−0.235∗∗∗	−2.604	−0.184∗∗∗	−2.99
LnTI	−0.045∗∗∗	−2.873	−0.051∗∗	−2.400	−0.037∗∗∗	−2.88
LnFI	0.135∗∗∗	2.984	0.152∗∗	2.452	0.146∗∗∗	4.23

**Table 9:** Driscol-Karaay Regression results (LCF dependent variable).

Variables	Driscoll-Kraay standard errors model	
	Coef.	Drisc/Kraay Std. Err.
LnREC	0.120∗∗∗	0.038
LnGDP	−0.184∗∗	0.070
LnTI	−0.037∗∗∗	0.011
LnFI	0.146∗∗∗	0.023
_cons	0.424	0.707

Note: ∗^,^ ∗∗^,^ and ∗∗∗ respectively, significance at the 10 %, 5 %, and 1 % levels.

Assessing the results on an individual level suggest that renewable energy portray a positive association with LCF at the 1 % significant level. In order words, a 1 % increase in renewable energy consumption would correspond to between 0.097 % and 0.120 % improvement in the LCF. The result is validated by the outcome of the robustness test (Table 9). Additionally, the finding is reinforced by Ali et al. [99] for the South American economic region, Pata [100] for the US and Japan, and Radmehr et al. [41] for the G7 bloc. This is an indication that the call for countries to migrate from non-renewable energy consumption to renewable energy sources is in the right direction as the latter ensure environmental progress. Also, the finding supports the EU's agenda to move from conventional options to renewable energy sources, stressing that such a movement is advantageous for environmental progress as it aligns with the region's Green Deal and its aim for Europe to become the first climate-neutral continent by 2050.

On the connection between economic progress and the load capacity factor, our result reveals statistically significant negative coefficients for economic growth at the 1 % and 5 % levels of significance, which are consistent throughout the results. This shows that economic growth exerts a destructive effect on environmental quality among the EU countries. The result emphasis the EU's challenge of finding an equilibrium between economic growth and environmental sustainability. More specifically, a variation of 1 % in economic growth imposing between 0.147 % and 0.235 % surge in environmental damage, necessitates the pursuit of sustainable growth pathways. This encompasses employing sterner regulations, investing in green technologies, and incentivizing environmental practices. This suggests that the EU should aggressively pursue its environmental policies in consonance with international agreements such as the Paris Agreement and the UN SDGs to ensure that public health, biodiversity, and ecosystems are protected. Although renewable energy use advance environmental progress, its proportion in the total energy mix of EU countries is only about 22 % [24]. This implies that economic progress among the EU largely relies on fossil fuels to power its industries which increases the carbon footprint among these economies, thus reducing environmental progress by lowering the LCF. It is, therefore, important that the EU countries increase the share of eco-friendly energy in the total energy consumption mix to boost economic expansion to reap a positive environmental outcome. This is consistent with the outcome of the robustness analysis and the works of Gyamfi et al. [101] and Shayanmehr et al. [41] but contradicts that of Ofori et al. [102].

Moving on now to consider the technology innovation and Load capacity factor connection, the findings reveal statistically significant but negative elasticities for technological innovation across the three models. The findings accentuate the region's challenge of ensuring that technological innovation compliments environmental sustainability, as a 1 % increase in the former accounts for a 0.037 %–0.051 % deterioration in environmental quality. Although this finding contravenes the expected outcome, it is plausible. We make the point that regardless of the constructive impact of technology innovation on environmental progress as found in the studies of Kirikkaleli et al. [103], the deviation of the result which aligns with Ali [86] could be explained based on three arguments. First, that new technology development usually involves the use of more energy to manufacture and operate and this may end up increasing the carbon footprint of a country, thereby reducing environmental quality. Second, technology innovation may lead to large amounts of electronic waste due to the fast pace at which electronic devices become obsolete, which if not well disposed of may degrade the environment. Third, in the installation of new technology infrastructure (e.g. power plants), natural habitats may be disturbed which may impact negatively on the environment. This requires policies that encourage eco-friendly technologies and sustainable research and development. This can be achieved by drawing parallels between technological progress and environmental sustainability via the integration of environmental deliberations into economic approaches, incentivizing eco-friendly innovation, and employing vigorous regulatory frameworks. Moreover, the promotion of corporate responsibility, public awareness, and international collaboration can support the region's quest as pacesetters in the global sustainability drive.

Finally, it is demonstrated that financial inclusion imposes a statistically significant positive effect on environmental quality under all the models at the 1 % and 5 % levels of significance. As expected, our robustness analysis also reveals a similar result. Thus, if financial inclusion increases by 1 %, environmental quality will improve between 0.135 % and 0.152 %. This highlights an important connection with practical implications for the EU. Improving financial inclusion tends to encourage sustainable economic practices by aiding wider access to financial capital for eco-friendly investments. This implies that EU policies and outward 10.13039/100005836FDI strategies should be targeted at advancing financial systems in member countries, supporting microfinance, digital banking, and inclusive financial services. By doing so, the EU can foster economic empowerment and environmental sustainability simultaneously, positioning itself as a leader in integrating financial inclusion with environmental policies.

### 4.5 The method of moments Quantile regression

The following part of this paper moves on to discuss the determinants of our covariates on various levels of load capacity factor, ranging from the 10th quantile to the 90th. The moment of quantile regression estimator was deployed to undertake this exercise. Table 10 presents the results of the study outcome. The result reveals that renewable energy exerts a positive impact on environmental quality via growth in the LCF at a 1 % level of significance across all quantiles. Interpretively, the expansion of the elasticity of clean energy by 1 % corresponds with a growth of the load capacity factor by 0.129 %, 0.125 %, 0.120 %, 0.116 %, and 0.112 % for the 10th to 90th quantiles respectively. Although renewable energy maintains its positive impact, it is observed that the magnitude of the effect decreases with increasing levels of the load capacity factor.

**Table 10:** Results of the Method of Moments Quantile regression (LCF dependent variable).

Variables	Location	Scale	Quantiles				
			0.10	0.25	0.50	0.75	0.90
LnREC	0.120∗∗∗	−0.005	0.129∗∗∗	0.125∗∗∗	0.120∗∗∗	0.116∗∗∗	0.112∗∗∗
LnGDP	−0.184∗∗∗	−0.009	−0.169∗	−0.176∗∗	−0.185∗∗∗	−0.192∗∗∗	−0.198∗∗∗
LnTI	−0.037∗∗	−0.003	−0.031	−0.034∗	−0.037∗∗	−0.040∗∗	−0.042∗∗
LnFI	0.146∗∗∗	0.013	0.124∗∗	0.134∗∗∗	0.148∗∗∗	0.158∗∗∗	0.168∗∗∗
_cons	0.424	0.129	0.219	0.314	0.442	0.541	0.627

Note: ∗, ∗∗, and ∗∗∗ respectively, significance at the 10 %, 5 %, and 1 % levels.

Furthermore, the study reveals that economic progress destroys environmental quality across all quantiles. Thus, with the negative effect, it is implied that environmental quality declines by 0.169 %, 0.176 %, 0.185 %, 0.192 %, and 0.198 % with a 1 % variation in economic progress under the various quantiles, respectively. In other words, economic progress is detrimental to environmental quality. A visual assessment of the result indicates that the destruction of economic progress on environmental advancement increases with higher levels of the LCF.

Concerning technology innovation, the result posits that the elasticities of technology innovation are significant for all the quantiles except the first quantile. Thus, the estimated elasticities of technology innovation are −0.031 %, −0.034 %, −0.037 %, −0.040 %, and −0.042 % under the 2nd to 9th quantiles respectively. This suggests that if technology innovation increases by 1 %, environmental quality decreases via a deterioration in the LCF by the percentages mentioned above. Similar to the absolute values of elasticities of economic progress, that of technology innovation increases with increasing levels of load capacity factor as represented by the various quantiles.

Lastly, the financial inclusion was recorded to positively induce load capacity factor under all the quantiles at 5 % and 1 %, respectively. The result indicates that whereas a variation of 1 % in financial inclusion would account for a 0.124 % increase in environmental quality in countries with low amounts of environmental progress, that of the countries with higher amounts of environmental advancement would increase by 0.168 %. The result are not in line with Shang [104]. While the findings support of [75, 105 and 106] but contradicts.

**Fig. 4:**
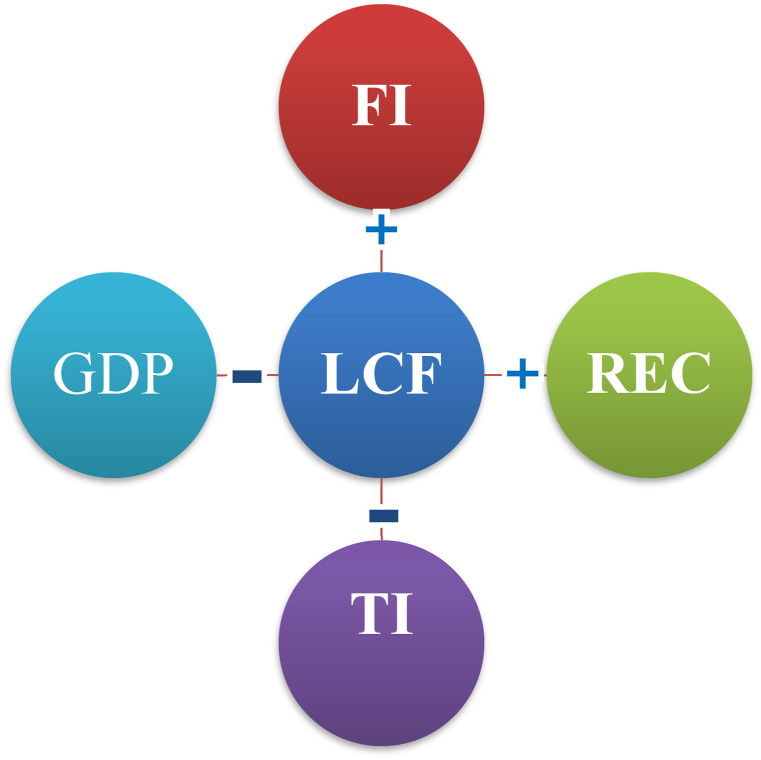
Graphical findings from MMQR

Here we make the argument that financial inclusion improves access to and use of clean energy. That is expanding access to affordable green financial options can help communities, organizations, and/or individuals to adopt environmentally friendly technologies such as energy-efficient appliances. By so doing, financial inclusion can have an indirect positive effect on environmental progress. Additionally, financial inclusion promotes environmental excellence by providing the required capital for individuals or organizations to venture into environmental startups that can help improve environmental quality in the long run (Khan and Rehan). Lastly, via financial inclusion, sustainable production technologies such as agroforestry, water-efficient irrigation systems, waste management and recycling systems can be developed to improve environmental quality. Fig. 4 shows the graphical findings from MMQR.

### 4.6 A comparison of econometric outcomes

**Fig. 5:**
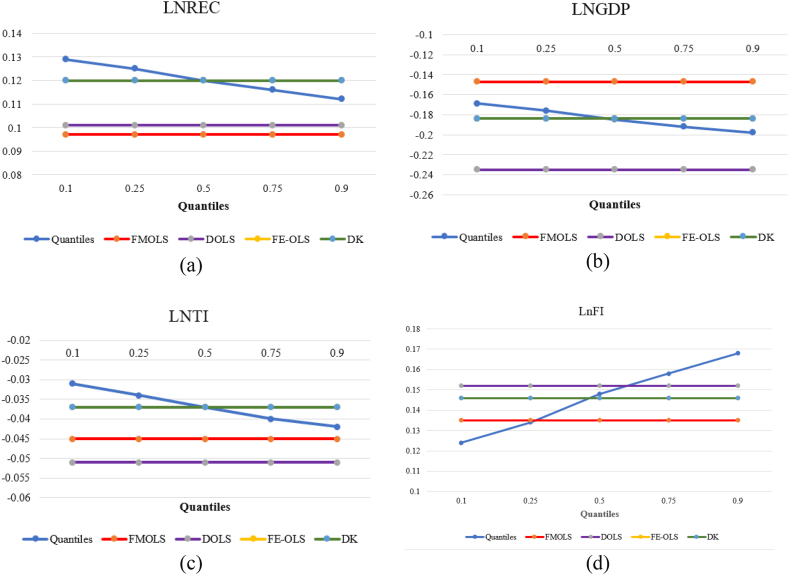
Comparison of coefficients of used econometric approaches (Quantiles, FMOLS, DOLS, FE-OLS, and KD). Panels (a), (b), (c), and (d) compare the coefficients estimated by models for the variables lnREC, lnGDP, lnTI, and lnFI, respectively.

Here we graphically compare and discuss the results obtained from the various estimation approaches adopted for the study. Thus in Fig. 5, the outcomes from all the estimation procedures are compared. It is observed that the results from the models except the MMRQ are static. Thus, the MMQR results are heterogeneous and dynamic across all quantiles. Focusing on the graphs of the individual factors, it is noted that the elasticities of renewable energy under the MMQR decline across the various quantiles but remain positive. Also, the elasticities of financial inclusion under the MMQR are seen to increase across the various quantiles in the positive zone. This suggests that eco-friendly energy and financial inclusion promote the surge in environmental progress which is consistent with the other results throughout the study. On the contrary, the elasticities of economic progress and technology innovation are seen to decrease across the various quantiles but in the negative zone. Thus, justifying our finding so far that economic growth and technological innovation promote environmental destruction. Furthermore, a careful examination of Fig. 5 reveals that while the FMOLS produces the lowest elasticities of renewable energy, it produces the highest for economic progress. Also, it can be seen that the DOLS produces the lowest elasticities of economic progress and technology innovation but produces the second lowest elasticities of renewable energy. Interestingly, the MMQR produced the highest elasticities for all the variables except economic progress.

### 4.7 Results of panel causality

To conclude, we investigate the long-run association between the study factors using the Granger causality test (Table 11). The result documents both one-way and the reciprocal movement between the factors. Specifically, a one-way movement has been established from eco-friendly energy to environmental quality and from technology innovation to environmental quality. On the contrary, a two-way movement is established from economic progress to environmental quality and vice versa and from environmental quality to financial inclusion and vice versa.

**Table 11:** Results of the Granger causality test.

Null hypothesis	W- bar	Z-bar	P-value	Causality	Diction
LnLCF ≠ LnREC	2.538	−0.452	0.650	No	Unidirectional
LnREC ≠ LnLCF	5.518	2.471∗∗	0.013	Yes	
LnLCF ≠ LnGDP	1.870	3.138∗∗∗	0.001	Yes	Bidirectional
LnGDP ≠ LnLCF	1.620	2.235∗∗	0.025	Yes	
LnLCF ≠ LnTI	2.608	−0.384	0.700	No	Unidirectional
LnTI ≠ LnLCF	5.481	2.434∗∗∗	0.014	Yes	
LnLCF ≠ LnFI	2.726	3.156∗∗∗	0.001	Yes	Bidirectional
LnFI ≠ LnLCF	2.834	3.393∗∗∗	0.000	Yes	

## 5 Conclusion and policy implications

### 5.1 Conclusion

The current research explores the association between financial inclusion, renewable energy consumption, technological innovation, and environmental sustainability in European countries from 2004 to 2018. This research expands the literature by examining the impact of financial inclusion on LCF in the context of European countries. In addition, the study employed the novel technique of moment quantile regression (MMQR). The MMQR model enables us to discover the various impacts of the exogenous factors across multiple quantiles of the conditional distribution of LCF. Likewise, the study utilized the Granger causality test to capture the causality interconnection among the selected variables.

The results from MMQR show that renewable energy increases the LCF in the European economies while economic growth causes a reduction in LCF in lower, median, and high quantiles. Therefore, improving the rate of renewable energy enhances ecological sustainability while economic progress reduces ecological quality. The elasticities of technological innovation decline across the various quantiles. Hence, LCF decreases as technological innovation expands in the European countries, showing that technological innovation is obstructing sustainable development in these countries. On the other hand, the findings from MMQR show that financial inclusion has positive and significant effects on LCF across all quantiles, indicating that financial inclusion plays a positive role in promoting ecological sustainability in these economies. This finding suggests that policymakers should use financial inclusion to reinforce ecological sustainability. Finally, the causality test findings approve a causal feedback interconnection among financial inclusion, economic growth, and LCF. Besides, the findings affirm a unidirectional causal from renewable energy to LCF. The reason for these findings is that technological development could generate more pollution-intensive industries. In this context, technology development usually involves the use of more fossil fuels, thereby reducing environmental quality. In contrast, greater financial inclusion has generated more green energy consumption; therefore, the government must continue to promote financial inclusion policies that are environmentally friendly.

### 5.2 Policy implications

The study's findings have some policy suggestions for EU countries.

First, the results of this research confirmed that FI contributes significantly to the improvement of environmental quality in the EU countries. Financial inclusion promotes load capacity factor in EU nations by encouraging the industry to implement sustainable manufacturing processes. Therefore, to boost the efficacy of 10.13039/100017146FI in eliminating environmental degradation, the financial system should not disburse funds for the production of non-environmental goods, and it ought to guarantee that these funds are invested in environmentally friendly projects. Hence, these economies should initiate green projects by financing them at low-interest rates to create a balanced financial system. In addition, additional funds can be allocated to R&D projects to develop green technology, improve energy efficiency, and consequently produce environmentally friendly goods. Secondly, the outcomes of the research showed that environmental quality is negatively and significantly impacted by technological innovation. This result is indicative of the fact that major technological advancement policies in European Union countries centered on energy-intensive technological options. Therefore, leaders of EU countries should implement novel energy policies to improve environmental quality through the development of green technological innovation. In fact, technology can play a key role in encouraging the use of renewable energy. On the other side, if innovations in technology are not directed toward sustainable innovations, it may result in further destruction of the environment. To avoid the negative environmental impact of technological innovation, governments in European countries should focus more on the development of environmentally friendly technologies and boosting the use of renewable energy. Finally, the negative impact of economic growth on the load capacity factor calls for a rethink among EU governments to take action to mitigate this adverse effect. In this context, it is recommended that EU countries include the aspects of quality and efficiency of development goals in the design of economic strategies.

### 5.3 Study limitations and directions for future research

This research only focused on European countries, and due to the non-availability of data, the timeframe is limited to 2018. Future studies can either focus on other economies and regions or use the latest data as they become available. Additionally, our study did not consider the individual outcomes of the EU panel studied. Given there could be divergent policies and features across these economies, their results may differ on an individual basis. Hence, future studies can appraise the country-specific dynamics of this subject matter in EU nations. Furthermore, future empirical studies can use other determinates of LCF, such as financial technology, green growth, energy aid, and structural change, among others. Lastly, this research uses the novel MMQR approach to capture the linkage among the tested variables. Future empirical studies can use other advanced panel approaches. Finally, future studies can address the linkage between financial inclusion and LCF in European countries by considering the STIRPAT model.

## CRediT authorship contribution statement

**Ahmed Samour:** Conceptualization. **Riza Radmehr:** Methodology, Formal analysis. **Ernest Baba Ali:** Formal analysis, Data curation. **Samira Shayanmehr:** Validation, Supervision. **Elvis Kwame Ofori:** Validation, Supervision. **Jana Ivanič Porhajašová:** Writing – original draft, Formal analysis. **Mária Babošová:** Writing – review & editing, Writing – original draft, Validation, Funding acquisition. **Miroslava Kačániová:** Writing – review & editing, Writing – original draft, Project administration. **Stephen Kelechi Dimnwobi:** Writing – original draft, Validation.

## Data availability statement

The data is publicly available on the following links: https://www.imf.org/en/Home https://data.worldbank.org/data.footprintnetwork.org/. databank.worldbank.org.

## Declaration of competing interest

The authors declare that they have no known competing financial interests or personal relationships that could have appeared to influence the work reported in this paper.

## Abbreviations

LCF: Load Capacity Factor
MMQR: Method of Moment Quantile Regression
EF: Ecological Footprint
SDG: Sustainable Development Goals
GHG: Greenhouse Gas
TI: Technology Innovation
ARDL: Autoregressive Distributed Lag
AMG: Augmented Mean Group
FI: Financial Inclusion
GDP: Gross Domestic Product
REC: renewable energy Consumption
CSD: Cross-Sectional Dependence
IPS: Im, Pesaran, & Shin
CIPS: Cross-Sectionally Augmented Im, Pesaran, & Shin
FE-OLS: Fixed Effects OLS
D-OLS: Dynamic-Ordinary Least Squares
FMOLS: Fully Modified-OLS
